# Results from the First Year of Implementation of CONSULT: Consultation with Novel Methods and Simulation for UME Longitudinal Training

**DOI:** 10.5811/westjem.2015.9.25520

**Published:** 2015-10-22

**Authors:** Keme Carter, Andrew Golden, Shannon Martin, Sarah Donlan, Sara Hock, Christine Babcock, Jeanne Farnan, Vineet Arora

**Affiliations:** * University of Chicago, Section of Emergency Medicine, Chicago, Illinois; † University of Chicago Pritzker School of Medicine, Chicago, Illinois; ‡ NorthShore University HealthSystem, Division of Emergency Medicine, Evanston, Illinois; § University of Chicago, Section of Hospital Medicine, Chicago, Illinois; ¶ University of Chicago, Section of General Internal Medicine, Chicago, Illinois

## Abstract

**Introduction:**

An important area of communication in healthcare is the consultation. Existing literature suggests that formal training in consultation communication is lacking. We aimed to conduct a targeted needs assessment of third-year students on their experience calling consultations, and based on these results, develop, pilot, and evaluate the effectiveness of a consultation curriculum for different learner levels that can be implemented as a longitudinal curriculum.

**Methods:**

Baseline needs assessment data were gathered using a survey completed by third-year students at the conclusion of the clinical clerkships. The survey assessed students’ knowledge of the standardized consultation, experience and comfort calling consultations, and previous instruction received on consultation communication. Implementation of the consultation curriculum began the following academic year. Second-year students were introduced to Kessler’s 5 Cs consultation model through a didactic session consisting of a lecture, viewing of “trigger” videos illustrating standardized and informal consults, followed by reflection and discussion. Curriculum effectiveness was assessed through pre- and post- curriculum surveys that assessed knowledge of and comfort with the consultation process. Fourth-year students participated in a consultation curriculum that provided instruction on the 5 Cs model and allowed for continued practice of consultation skills through simulation during the Emergency Medicine clerkship. Proficiency in consult communication in this cohort was assessed using two assessment tools, the Global Rating Scale and the 5 Cs Checklist.

**Results:**

The targeted needs assessment of third-year students indicated that 93% of students have called a consultation during their clerkships, but only 24% received feedback. Post-curriculum, second-year students identified more components of the 5 Cs model (4.04 vs. 4.81, p<0.001) and reported greater comfort with the consultation process (0% vs. 69%, p<0.001). Post- curriculum, fourth-year students scored higher in all criteria measuring consultation effectiveness (p<0.001 for all) and included more necessary items in simulated consultations (62% vs. 77%, p<0.001).

**Conclusion:**

While third-year medical students reported calling consultations, few felt comfortable and formal training was lacking. A curriculum in consult communication for different levels of learners can improve knowledge and comfort prior to clinical clerkships and improve consultation skills prior to residency training.

## INTRODUCTION

Medical errors in the inpatient setting are frequently attributed to breakdowns in communication; as many as 70% of errors are attributed to communication errors.^1^–^6^ One type of communication that is especially common in emergency department (ED) care is the consultation, whereby one provider seeks formal recommendations from another provider regarding the care of a patient; 40% of all ED visits require at least one consultation by Emergency Medicine (EM) providers.^4^,^7^ There is increasing recognition that a formal approach to requesting consultations is necessary to prevent communication errors from occurring.^8^ Curbside consultations, or unstructured consultations, whereby a consultant is asked to provide recommendations regarding the care of a patient without formal assessment and communication, have historically been a common practice in medicine.^8^ However, when compared to formal consultations, curbside consultations can adversely affect patient care.^8^

There is a large gap of knowledge on effective consultation education amongst trainees and practicing physicians. Previous research focused on providing an “educational protocol” for medical students to utilize while working in the ED, including pre-developed scripts and checklists when requesting specialty consultation.^9^ However, many students and trainees still receive little to no formal education specifically on consultation. A significant body of research on consultation has been developed within the specialty of EM. A conceptual model developed by Kessler et al, the “5 Cs of Consultation”, has been proposed to describe a standardized consultation from the ED to hospital-based services.^10^,^11^ The 5 Cs include Contact, Communicate, Core Question, Collaboration, and Closing the Loop, and offers specific action items for each component (Appendix A). This model has been tested and validated in a randomized controlled trial amongst EM residents and increased effectiveness of consult communication in this setting.^12^,^13^ To our knowledge, the 5 Cs model has not been implemented into a formal undergraduate medical educational curriculum on consult communication.^4^ Because the practice of learning consultation communication through single point repetition may not result in improvement of this skill, experts in the field believe that formal training in consultation communication should exist at various levels of training, including undergraduate medical education.^4^,^13^

The specific aims of this study were to develop, pilot, and evaluate the effectiveness of a consultation communication curriculum based on Kessler’s 5 Cs model for different learner levels in undergraduate medical education that can be implemented as a longitudinal curriculum. First, we aimed to conduct a targeted needs assessment among third-year medical students on their experiences, comfort level, and instruction in calling consultations. Then, based on these results, we aimed to introduce and evaluate a curriculum in calling consultations during one academic year for second-year and fourth-year medical students. Second-year students received instruction on standardized consultation communication, and fourth-year students received a didactic and simulation based curriculum that enabled structured practice of consultation skills (Appendix B). We hypothesized that this curriculum would improve consultation communication knowledge, skills, and attitudes of the target learner groups.

## METHODS

### Targeted Needs Assessment

To determine the need for a formal consultation curriculum, an eight-question, paper-based, anonymous targeted needs assessment was developed internally by the authors using literature review and expert opinion. The survey was administered to third-year students at the conclusion of their clinical rotations in the Spring of 2013. The survey asked questions in four general areas: [1] previous education on calling consultations, [2] previous exposure to calling consultations, [3] current comfort level of requesting consultations and collaborating with consultants, and [4] identification of the five components to be included in effective consultations according to Kessler’s 5 Cs model. The survey included yes/no questions, such as “Have you ever been instructed on how to call a consult?”, as well as questions in which participants used a five point Likert-type scale, for example, to rate their “comfort requesting a consult” from Very Comfortable to Very Uncomfortable (Appendix C).

### Curriculum Design and Implementation

#### Second-Year Medical Students

In a lecture hall setting, as one group, second-year students participated in a 50-minute consultation communication didactic during the 2014 winter quarter of a required clinical skills course. Prior to participating, all students had completed at least 30 hours of required clinical observation experiences. The authors filmed two trigger tapes for the didactic session. The first trigger tape demonstrated a curbside consultation and illustrated patient safety concerns that can arise with informal consultations. After discussion of the first trigger tape and the behaviors that led to poor consultation communication, the students were instructed on Kessler’s 5 Cs model through a didactic lecture developed for all learner groups. Students then viewed the second trigger tape that illustrated a consultation that followed the 5 Cs model, and participated in a discussion of the elements of this standardized consultation that led to a successful collaborative relationship between the provider and consultant.

#### Fourth-Year Medical Students

Fourth-year students participating in the required, month-long EM clerkship during the 2013–2014 academic year were consented for participation. Oral consent was obtained from July 2013 through April 2014. Up to twelve students per month participate in the EM clerkship, which includes 15 hours in dedicated didactic, simulation, and educational time.

**Figure 1:**
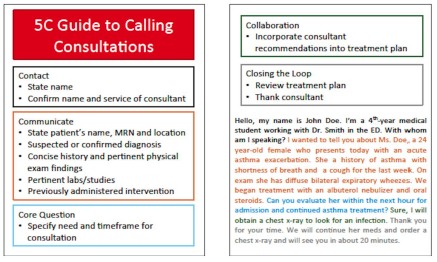
5 Cs pocket card given to students participating in the consultation curriculum. *MRN,* medical record number

The curriculum was comprised of three parts: the didactic portion, consultation communication practice during high-fidelity simulations, and structured debriefing. A 30-minute didactic lecture was given by the EM Clerkship Director, emphasizing the importance of consultation communication and instructing students on the 5 Cs model. Following the lecture, students were provided with a pocket card detailing Kessler’s 5 Cs model as an added tool for reference during their simulation sessions and clinical work, including detailed information to be included in each section of a consultation (Figure 1). During the simulation sessions, students called consultations that were recorded and reviewed by EM attending physicians who were trained using the 5 Cs Model. High-fidelity simulation was chosen as a teaching method to give students a realistic and engaging experience, as prior work has shown that although high-fidelity simulation can trigger a “stressful” response, EM trainees continue to desire to participate in future sessions.^14^,^15^ The students then received structured feedback on their consultation communication performance during the debriefing period. Cases used in this study were selected on the basis of common presentations to the ED and included gastrointestinal bleed, myocardial infarction, ectopic pregnancy, urosepsis, diabetic ketoacidosis, aortic dissection, status epilepticus, hyperkalemia, and symptomatic bradycardia.

### Curriculum Evaluation

#### Second-Year Medical Students

Second-year students participating in the consultation communication didactic completed a pre- and post-curriculum survey. Both surveys assessed the students’ knowledge of the consultation process with yes/no statements such as “I understand what a formal consultation is” as well as a multiple choice question asking them to “select the 5 components of a consultation that have been shown to improve consultation communication” according to Kessler’s 5 Cs model. Both the pre- and post-curriculum surveys asked the students to “rate their level of comfort requesting a consultation given the necessary medical background.” Additionally, the post-curriculum survey assessed overall satisfaction with the consultation didactic.

#### Fourth-Year Medical Students

Throughout the month-long clerkship, fourth-year students participated in three simulation sessions (one session per week). The initial, or baseline, simulation experience occurred prior to the implementation of the didactic component of the curriculum, and the students underwent pre- and post-curriculum evaluations. Consultation communication skills during simulation were measured by EM attending physicians who were trained using Kessler’s 5 Cs Model Checklist for Assessing Physician Consultations and the Global Rating Scale (GRS) for Assessing Physician Consultations (Appendix A and D). Kessler’s 5 Cs Model Checklist was adapted from a business consultation model by an expert panel and validated in a cohort of EM and EM/internal medicine residents.^12^ The checklist included 13 different components that should be included in effective consultations, such as specifying the need for a consultation. The checklist components were valued as “Done” or “Not done.” Kessler’s GRS tool was developed through literature review and expert panel recommendations followed by review and modification by consultants.^13^ The GRS utilized a five point Likert-type scale from “Not effective” to “Extremely effective” for seven items, such as patient case presentation, to indicate perceived efficacy of the consultation by the attending physician.

Each consultation was rated by two independent evaluators per consultation using the GRS and the 5 Cs Checklist. The scores for each component of the evaluations were averaged for each consultation, creating a composite, single evaluation per consultation performed.

Learner satisfaction with the fourth-year curriculum was assessed through use of a ten-question survey completed by students at the end of the EM clerkship. Learners were asked to rate the value of each curriculum component including the didactic lecture, simulated cases, and pocket cards. All survey questions were rated on a five point Likert-type scale from “Strongly disagree” to “Strongly agree.”

### Data Analysis

Data from the fourth-year curriculum was collected from July 2013 to April 2014. For each consultation, the 5 Cs checklist was recorded with the completion of individual checklist items, as well as having a proportion of the 12 points completed (i.e. 7/12). Responses gathered from the needs assessment, the GRS, and the learner satisfaction survey were translated into ordinal numbers for data analysis (i.e. 1=“Not effective”, 5=“Extremely effective).

Evaluations were compared for the pre- and post-curriculum simulations. For the checklist, the proportion of inclusion of each component and the absolute difference between values before and after curriculum implementation were reported. For the GRS, the averages and standard error of the means were reported, as well as the absolute differences in these values before and after curriculum implementation. Changes in the average checklist completion and GRS values pre- and post-curriculum implementation were analyzed using two-tailed, unpaired Student’s t-tests to examine if the curriculum was overall successful at increasing thoroughness and efficacy of consultations called. Rater agreement was analyzed by calculating the distribution of differences between the two evaluators.

All statistical analyses were performed using Stata 12.1 (StataCorp, College Station, TX). A p-value of less than 0.05 was considered statistically significant in all analyses. This study was approved by the appropriate Institutional Review Boards.

## RESULTS

### Targeted Needs Assessment

**Figure 2:**
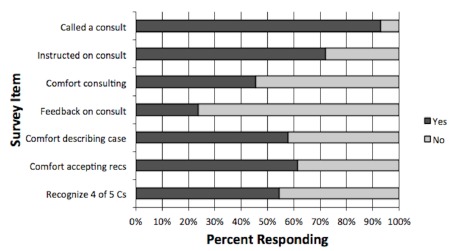
Percentage of students answering “yes” and “no” to each survey item of the targeted needs assessment.

A total of 57 third-year students out of an eligible 96 completed the targeted needs assessment, resulting in a 59% response rate. As shown in Figure 2, 53 (93%) of third-year students completing the survey reported calling a consultation during any of their third-year rotations. Thirteen students (24%) reported receiving feedback on their ability to call consultations from their supervising resident or the recipient of the consult. Forty-one students (72%) reported receiving instruction on how to call a consult, and of those, almost all (40/41, 98%) described informal instruction by a resident during their third-year rotations.

Although most students reported calling consultations, less than half (26 students, 46%) were comfortable requesting a consultation. A higher fraction felt comfortable telling the patient’s story to a consultant (33 students, 58%) and receiving recommendations from a consultant (35 students, 61%). On the knowledge-based portion of the survey, roughly half (31 students, 54%) were able to correctly identify at least 4 of the 5 Cs of Kessler’s model. We used this data to demonstrate the need for inclusion of formal consultation communication training in the undergraduate medical education curriculum.

### Second-Year Medical Student Curriculum

Twenty-five second-year students completed the pre-curriculum survey, and 26 completed the post-curriculum evaluation. After receiving the curriculum, students were able to identify significantly more correct components of the 5 Cs model (4.04 vs. 4.81, p<0.001). The number of students understanding the definitions of formal and curbside consultations also significantly increased (24% vs. 100%, p<0.001; 16% vs. 100%, p<0.001; respectively). After the curriculum, more students indicated they would be comfortable in requesting consults (0% vs. 69%, p<0.001), while fewer felt they would need guidance while calling a consultation (60% vs. 31%, p<0.001). Learner satisfaction was very high, with 100% of students reporting that they were “Satisfied” or “Extremely satisfied” with the consultation didactic session.

### Fourth-Year Medical Student Curriculum

In the fourth-year curriculum, 117 students called 170 total simulated consultations–84 prior to receiving the curriculum and 86 after curriculum implementation. Each consultation was evaluated using the GRS and the 5 Cs checklist by two independent raters per consultation for a total of 340 evaluations. Analysis of the GRS evaluations showed that in each category, evaluators differed by 2 or more points on the Likert-type scale in less than 29% of consult evaluations and were in complete agreement or differed by 1 point on the scale in each category in 72–90% of evaluations. Analysis of the 5 Cs checklist showed that evaluators differed by 2 or more criteria in each category in less than 11% of consult evaluations and were otherwise in complete agreement or differed by 1 criterion in each category in 90–100% of evaluations. The scores for each component of the evaluations were averaged for each consultation, creating 170 composite evaluations.

**Figure 3:**
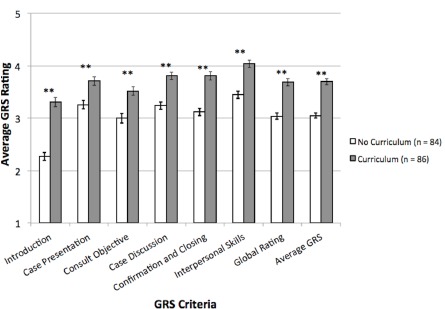
Global Rating Scale (GRS) assessment of consultation efficacy in seven criteria and an average of all criteria. Five point scale responses were converted to ordinal numbers where 1=“Not effective” and 5=“Extremely effective”. Means are graphed before and after curriculum implementation with standard error of the mean error bars. **p<0.001.

As shown in Figure 3, when compared to pre-curriculum consultation evaluation, the combined average score of the criteria measured in the GRS, or Average GRS, increased significantly following the implementation of the curriculum (3.05 vs. 3.70, p<0.001). Additionally, consultations performed after the implementation of the curriculum scored significantly higher in all seven individual criteria on the GRS (p<0.001 for all).

**Figure 4:**
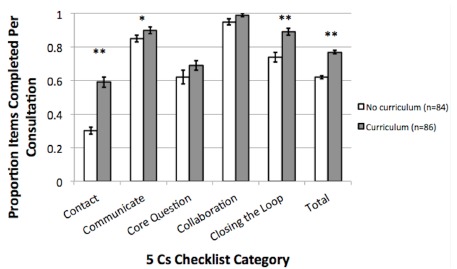
Proportion of 5 Cs checklist items completed per consultation in each category and total completion. For before and after the curriculum implementation, proportions are graphed with standard error of the mean error bars. *p<0.05. **p<0.001.

After receiving the curriculum, students completed higher proportions of the 5 Cs checklist compared to their consultation evaluations prior to receiving the curriculum (Figure 4). The overall checklist completion increased significantly (62% vs. 77%, p<0.001); specifically, the Contact (30% vs. 59%, p<0.001), Communicate (85% vs. 90%, p<0.05), and Closing the Loop (74% vs. 89%, p<0.001) sections had significantly higher completion by the students after the implementation of the curriculum.

Sixty-nine (59%) of the 117 fourth-year students who received the curriculum completed the learner satisfaction survey. Participants reported a high degree of satisfaction with the curriculum, as 99% (68/69) indicated being “Satisfied” or “Extremely satisfied.” When asked about the content of the curriculum, 100% of the students “Agreed” or “Strongly Agreed” that the information presented had not been taught previously in their medical education. About 94% (65/69) of students reported the simulation experiences helped prepare them for calling consultations in a clinical setting. Almost all (67/69, 97%) students rated the pocket card as useful. Finally, after the implementation of the curriculum, 94% (65/69) of students “Agreed” or “Strongly Agreed” with the statement, “I feel comfortable calling a consultation.”

## DISCUSSION

Through our curriculum, second-year students are introduced to the formal consultation process, and instructed on a standardized consult model prior to beginning clinical clerkships. Fourth-year students are instructed on the 5 Cs model, given a pocket card that encourages continued adherence to the standardized consultation, and practice their consultation skills in a structured, simulation setting. After participating in our consultation curriculum, second-year students demonstrated an increase in knowledge and understanding of the standardized consultation process and, although not surprising given their lack of significant clinical experience, reported higher levels of comfort with requesting a consultation given the necessary medical background. Fourth-year students scored higher on evaluations that assess thoroughness and perceived efficacy of consultation communication and reported higher levels of comfort in calling and discussing consultations compared to responses gathered in the targeted needs assessment. These results suggest that on-the-fly instruction in consultation communication is not adequate, and a formal curriculum is needed to improve skills and comfort level.

These findings have implications for the inclusion of additional educational interventions in undergraduate medical education to enhance the efficacy of consultation communication. This consultation communication curriculum, which can be implemented as a longitudinal experience, is novel and allows students to receive instruction prior to the third-year clerkships. It also reinforces this critical skill in the controlled environment of the simulation laboratory during a fourth year capstone experience. Our curriculum aligns well with the need for medical schools to address whether students are proficient in the Core Entrustable Professional Activities for Entering Residency, particularly those emphasizing the importance of interprofessional collaboration, understanding one’s role as a medical team member, and seeking help when necessary,^16^ which our curriculum specifically addresses. Although the 5 Cs Model was validated in a cohort of resident physicians, we believe our consultation curriculum is feasible, and was shown to be effective and suitable for this level of learner.

Future directions in assessing the effectiveness of this consultation curriculum include evaluating long-term retention of consultation communication knowledge and skills in a group of learners who receive the entire longitudinal curriculum. We plan to study consultants’ perceptions of consults called by students to determine if these skills translate to the clinical setting. Also, we plan to compare the consultation communication skills of interns who have received the longitudinal curriculum as medical students to entering interns who have not received consultation training, allowing for a control group study.

## LIMITATIONS

There are limitations to this study. Our research and educational curriculum were performed at a single site, and thus results may not be generalizable to other institutions. The response rate for the targeted needs assessment and fourth-year learner satisfaction surveys was 59%. As the surveys were anonymous, we were unable to follow up with students who did not complete the surveys to increase the response rate. Additionally, the analysis of consultations occurred only in controlled environments. In real clinical settings, the measures of an effective consultation according to Kessler’s model may be sacrificed for issues of timeliness, and other responsibilities of serving on a care team. Finally, our study describes interventions at the second and fourth-year levels. During year 1 of curriculum implementation, we were unable to study the same learners over time to determine if receiving the entire curriculum has any long-term educational benefit, but plan to do so in the future.

## CONCLUSIONS

Medical students are calling consultations during third-year clerkships, but formal instruction is rare. Developing and implementing longitudinal consultation curricula, with a didactic during the pre-clinical curriculum and simulation-based instruction during the EM clerkship, can help address the current deficit in undergraduate medical education and better prepare students to call consults before beginning clinical clerkships and prior to entering residency training.

## Supplementary Information
