# Supplementary material for: Results from the First Year of Implementation of CONSULT: Consultation with Novel Methods and Simulation for UME Longitudinal Training
Source: West J Emerg Med. 2015 Oct 22;16(6):845–50. doi: 10.5811/westjem.2015.9.25520 (PMC4651580; doi:10.5811/westjem.2015.9.25520)
Supplement: Supplementary file 1 [file wjem-16-845-s001.pdf]

Appendix A

5Cs Model Checklist for Assessing Physician Consultations

The five Cs and explanations	Checklist item	Done	Not done
Contact<br>Introducing the consulting and consultant physicians. Building the relationship.	- States name<br>- States rank and service<br>- Identifies supervising attending<br>- Identifies name of consultant physician	_____<br>_____<br>_____<br>_____	_____<br>_____<br>_____<br>_____
Communicate<br>Giving a concise story and asking focused questions.	- Presents a concise story<br>- Presents an accurate recount of information/case detail<br>- Speaks clearly	_____<br>_____<br>_____	_____<br>_____<br>_____
Core question<br>Preparing a specific question for request of the consultant. Deciding on reasonable timeframe for consultation.	- Specifies need for consultation<br>- Specifies timeframe for consultation	_____<br>_____	_____<br>_____
Collaboration<br>Planning a course of action that results from the discussion between the consulting physician and the consultant, including any alteration of management or testing	- Is open to and incorporates consultant's recommendations	_____	_____
Closing the loop<br>Ensuring that both parties agree to the plan and to maintaining proper communication about any changes in the patient's status.	- Reviews and repeats patient care plan<br>- Thanks consultant for consultation	_____<br>_____	_____<br>_____
