# Supplementary material for: Results from the First Year of Implementation of CONSULT: Consultation with Novel Methods and Simulation for UME Longitudinal Training
Source: West J Emerg Med. 2015 Oct 22;16(6):845–50. doi: 10.5811/westjem.2015.9.25520 (PMC4651580; doi:10.5811/westjem.2015.9.25520)
Supplement: Supplementary file 2 [file wjem-16-845-s002.pdf]

Appendix B

<table>
<tr><th colspan="6">Year 1 of Implementation of Consultation Curriculum</th></tr>
<tr><th>Spring 2013</th><th rowspan="3">Start of 2013-2014 Academic Year</th><th>Summer 2013</th><th>Autumn 2013</th><th>Winter 2014</th><th>Spring 2014</th></tr>
<tr><td>Targeted needs assessment completed by third-year medical students (class of 2014)</td><td colspan="4">Forth year medical students (class of 2014): Consultation Curriculum during Emergency Medicine Clerkship</td></tr>
<tr><td></td><td colspan="2"></td><td>Second-year medical students (class of 2016): Consultation curriculum during Clinical Skills Course</td><td></td></tr>
</table>
