# Supplementary material for: Results from the First Year of Implementation of CONSULT: Consultation with Novel Methods and Simulation for UME Longitudinal Training
Source: West J Emerg Med. 2015 Oct 22;16(6):845–50. doi: 10.5811/westjem.2015.9.25520 (PMC4651580; doi:10.5811/westjem.2015.9.25520)
Supplement: Supplementary file 3 [file wjem-16-845-s003.pdf]

Appendix C: Survey of Third-Year Medical Students

For this survey, please consider your experience calling consults during your third-year clerkship rotations.

During your third-year clerkships, have you ever called a consult service to request a consult for your patient?

☐1 Yes ☐2 No

Have you ever been instructed on how to call a consult?

☐1 Yes ☐2 No

If you marked YES, please indicate where you received this instruction (check any that apply):

☐ Clinical skills course
☐ Attending on inpatient clerkship service
☐ Resident or intern on inpatient clerkship service
☐ Sub-intern on inpatient clerkship service
☐ Fellow third-year medical student classmate
☐ Attending, fellow or resident on consult service
☐ Another health care provider (e.g., nurse practitioner, physician assistant, RN, etc.)
☐ Other – please specify: ____

Have you ever received feedback on your performance after calling a consult?

☐1 Yes ☐2 No

**Please rate your comfort with communicating with consult services below:**	*I feel very comfortable doing this and can perform it with ease.*	*I feel comfortable doing this.*	*I can do this, but appreciate guidance from other team members.*	*I feel uncomfortable doing this and would prefer not to do so.*	*I feel very uncomfortable doing this and cannot do it.*
Requesting a consult	☐1	☐2	☐3	☐4	☐5
Telling the patient's story and asking directed questions of a consult service	☐1	☐2	☐3	☐4	☐5
Receiving recommendations from a consultant	☐1	☐2	☐3	☐4	☐5

<u>Please select five</u> components of calling consultations that have been shown to improve consultation communication in patient care:

☐ Calling a consult in a timely manner (i.e., before 10am)
☐ Preparing a specific question for request for the consultant with an expected timeline
☐ Telling a concise presentation and asking focused questions
☐ Providing a 24-hour virtual pager that is always covered for consultation requests
☐ Closing the loop between both parties to ensure all agree to the plan
☐ Avoiding the use of non-physician providers in consult communication
☐ Introducing consulting and consultant physicians and establishing roles to build the relationship
☐ Collaborating to plan a course of action that results from discussion between both parties
☐ Involving the patient in shared decision-making about whether or not a consult is required
