# Supplementary material for: Results from the First Year of Implementation of CONSULT: Consultation with Novel Methods and Simulation for UME Longitudinal Training
Source: West J Emerg Med. 2015 Oct 22;16(6):845–50. doi: 10.5811/westjem.2015.9.25520 (PMC4651580; doi:10.5811/westjem.2015.9.25520)
Supplement: Supplementary file 4 [file wjem-16-845-s004.pdf]

Appendix D

Global Rating Scale (GRS) for Assessing Physician Consultations

Performance characteristic	Rating				
Introduction of involved parties	1 Not effective	2 Somewhat effective	3 Effective	4 Very effective	5 Extremely effective
Patient case presentation	1 Not effective	2 Somewhat effective	3 Effective	4 Very effective	5 Extremely effective
Specified consultation objective	1 Not effective	2 Somewhat effective	3 Effective	4 Very effective	5 Extremely effective
Case discussion	1 Not effective	2 Somewhat effective	3 Effective	4 Very effective	5 Extremely effective
Confirmation and closing	1 Not effective	2 Somewhat effective	3 Effective	4 Very effective	5 Extremely effective
Interpersonal skills	1 Not effective	2 Somewhat effective	3 Effective	4 Very effective	5 Extremely effective
Global rating	1 Not effective	2 Somewhat effective	3 Effective	4 Very effective	5 Extremely effective
